# Tuberculosis and Pulmonary Phthisis

**Published:** 1868-09

**Authors:** E. Boisseau

**Affiliations:** Professor Agrégé at Val-de-Grace


### Miscellaneous.

### Tuberculosis and Pulmonary Phthisis.—A Critical Review.

By E. BOISSEAU, Professor Agrégé at Val-de-Grace. Translated from the Archives Générales de Médecine, by J. H. Douglass, M. D., of New York.

We should not be surprised at the great number of works upon pulmonary phthisis which have appeared during the last few years, for it was very evident that the subject had by no means been exhausted; that more than one blank remained to be filled, more than one doubtful point to be elucidated.

In fact, what do we find in classical books upon *pulmonary phthisis?* What else than a pathological anatomy based upon the fact that tubercle is always a primordial lesion, from which all the rest are derived; a semeiology beyond criticism, but an etiology faulty, in which are found causes most incongruous and often very insignificant; hypotheses upon the intimate nature of the disease; a prognosis always fatal; a prophylaxis without any solid foundation, and consequently without influence; and, finally, a treatment given up to all the vagaries of empiricism?

We are inevitably brought, then, to renew our studies of this terrible disease, which Laennec seems to have taught us to recognize with greater certainty, only to prove to us the better how powerless we were.

The microscope had rendered science too great services not to be employed upon this subject. Hence doctrines founded upon pathological anatomy soon arose, which only had the effect of destroying the work of Laennec, reducing it to an hypothesis. For more than forty years it was considered a well-established fact that tubercle constituted in all cases the anatomical characteristic of pulmonary phthisis, and now this fact is attacked upon all sides. From the same means of investigation different conclusions have been drawn, so that the question, instead of being cleared up and simplified, has become complicated and obscure. The result has been that, while attempting to demonstrate more clearly the unity of the disease, it has been placed in doubt. By restricting the name of tubercle to gray granulation, by eliminating from tuberculous products everything which was not granulation, the tuberculous nature of phthisis has been contested when the type element has not been found. When it was stated that the caseous products were not special to the degenerating metamorphosis of the granulations, it was thought that they might be considered as independent of them, even when granulations and the caseous products were found together. Hence sprang all the new doctrines.

According to the Strasbourg school, there are two distinct kinds of phthisis:—1. The connective tuberculous phthisis of Laennec; 2. The epithelial, caseous phthisis, tuberculide.

Niemeyer, in his "Clinical Lectures," admits three principal forms:—1. Phthisis resulting solely from pneumonic processes; 2. Phthisis consisting from the beginning of a tuberculosis of the lungs; 3. Phthisis consisting primarily of a caseous pneumonia, and consecutively of a tuberculosis.

Finally, in a recent work, M. Ch. Bouchard seems disposed to admit four forms:—1. Tuberculosis without caseous pneumonia; 2. Caseous pneumonia without tuberculosis; 3. Tuberculosis primitively and caseous pneumonia consecutively; 4. Caseous pneumonia primitively and tuberculosis consecutively. It would be difficult to be more eclectic, and yet we are still far from the fourteen forms of Portal, the sixteen of Morton, the twenty of Sauvages.

The innovators have all formularized their doctrines from data furnished by pathological anatomy and by practice, and it is by placing ourselves in this double point of view, that we in our turn shall study, or rather discuss the question.

In order to limit the debate as much as possible, we can, from the pathological point of view, reduce the problem, as follows:—Are tubercular granulation and caseous pneumonia independent of each other, and if there are any relations between these two products, what are they? And, first, do all agree, even now, as to what is understood by tubercle? For the German and for most French micrographs, granulation remains the type, but caseous transformation being one of the normal phases, fatal even to the evolution of granulation, it would not be altogether reasonable to take from it the name of tubercle, even when it has undergone this metamorphosis. To restrict, then, the name of tubercle, as Virchow has done, to gray granulation, does not seem to me possible, and to make of this granulation a special product, absolutely distinct from yellow tubercle, as M. Empris has done, does not appear to me admissible.

Gray granulation, it is necessary to say, is not the only product capable of undergoing caseous transformation; there are others of a distinctly different nature (tubercles of glanders, syphilitic, gummy tumors, cancerous tumors, hæmorrhagic infarctus, etc.,) which can pass through identically the same transformations.

When Lebert believed he had discovered the tuberculous globule, the difficulty would have been solved had he not been the victim of an illusion; but it still exists, and we possess no criterion which enables us to distinguish a caseous tubercle from a miliary caseous nodosity of an inflammatory origin.

This being stated, what, then, do we find in the lungs of persons who die of chronic pulmonary phthisis? More or less extensive caverns, a greater or less amount of caseous products, and often also, granulations.

MM. Herard and Cornil declare that they found, in all cases in which they made an autopsy, tuberculous granulations and also caseous broncho-pneumonia.

M. Villemin, who believes tubercles may be present in the lungs under three forms—granulations, nodules and infiltrated masses—found two forms of the process, or even all three at a time.

If we judge from the little value Niemeyer gives to the chronic pneumonic processes which, he says, ordinarily show a well-marked tendency to recovery, he has not often made the autopsy of persons who have died of simple caseous pneumonia. Most of them had the misfortune to become tuberculous, since it, according to him, is the greatest danger which threatens those afflicted with phthisis.

Although tubercles are for Lebert more frequently the consequence than the cause of chronic disseminated pneumonia (caseous phthisis of authors,) he does not doubt the frequent coëxistence of granulations and caseous products.

Consequently we can say with a great number of authors: *at the autopsy of those who have died from phthisis, we ordinarily find both granulations and caseous masses.*

M. Ch. Bouchard, generally agreeing with the Strasbourg school, is of a contrary opinion. According to this observer, we often find consumptives who succumb rapidly to the destructive processes of the lungs, or who die after having carried caseous masses for years, and in whose lungs no miliary granulation can be found. *But it is impossible to deny as it is impossible to prove anatomically that a mass of caseous pneumonia had primitively as centre a gray granulation, the two products being capable of fusing into one common caseous mass.*

In chronic disseminated pneumonia from mechanical cause, of Lebert, the charcoal or some other particle is present, and if in chronic disseminated pneumonia without mechanical cause (phthisis of authors,) we do not find granulations, we are not on that account authorized to deny that they never existed. The persistence of a cause is not indispensable to the acknowledgment of its effects.

Were it not that this fact is of a nature to suggest difficulties, I should no longer consider it, because that similarity of appearances which at a given moment exists between caseous pneumonia and tuberculous granulation led Laennec into error.

I agree with Lebert that undoubted tubercles found in other organs than the lungs are not sufficient to solve the question, and I shall confine my examination to those cases in which granulations and caseous products coëxist in the lung itself, cases which, as we have seen, are the most common.

The question in dispute is, then, reduced to the following:—Are the miliary nodules which are met with in the midst of pneumonic processes the distinct inflammatory products of tuberculous granulations? M. Charles Bouchard does not doubt it, and he thinks Drs. Herard and Cornil have called that *tuberculous* which should have been called *inflammatory.*

All diffused masses quite separated from each other, without trace of degeneration towards the centre, are considered by Dr. Bouchard as a simple inflammatory product; granulation, besides its seat, form, and volume of its elements, being characterized by the very close grouping of the nuclei of which it is composed, their fatal and very rapid tendency to mortification.

The globular cells with nuclei, which are formed in the nodules, can attain from $0^{mm},012$ to $0^{mm},015$, while those of granulations do not exceed $0^{mm},008$. Regarded as to dimensions, a few thousands of a millimetre is all the difference between the cells.

These characteristics, "without which there are no points of departure," have not appeared sufficient to most micrographists. Lebert declares that the microscope gives no satisfactory results.

M. Villemin, examining these miliary nodosities at the commencement of the process, has found elements absolutely identical in form, dimensions or other characteristics with those which are developed in the proliferating zone of tubercles of the serous, of the mucous membranes, etc. Yet, what are the anatomical elements which proliferate to produce inflammatory nodosities? The vesicular epithelium, according to the partisans of caseous, epithelial phthisis.

But M. Villemin, in an article published in the *Archives Generales des Medecine* for 1866, has demonstrated that the existence of this epithelium is very problematical, and Mandl, since 1857, has arrived at about the same conclusions. Lebert says he has sought in vain for it in the adult. At the International Congress, Badoky, of Pesth, presented some very beautiful preparations tending to demonstrate the non-existence of this epithelium.

If this opinion is correct, and for reasons too numerous to give here, I am quite inclined to think it is, it is impossible to attribute miliary nodosities to the proliferation of the vesicular epithelium. But, even admitting the existence of this epithelium, the cause of caseous phthisis does not appear to be strengthened by it.

Lebert, who regards inflammation of the aveoli (alvéolite,) otherwise called vesicular pneumonia, as the most frequent anatomical form of pulmonary phthisis, is far from making this pneumonia consist in the simple proliferation of an epithelium, the existence of which has, moreover, never been positively established. The proliferation of the connective tissue also has its part as well as the proliferation of the epithelium, and the interstitial and peribronchial connective tissue furnish elements entirely like those of true tubercle, true tuberculous granulations, if they can be judged of by microscopic examination. But the Professor of Breslau denies their tuberculous character, because they are surrounded by a connected irritation of a diffuse interstitial pneumonia.

As no one has ever denied that tuberculous granulations could provoke about them precisely the same irritation, this differential characteristic appears to me, then, insufficient.

Lebert, as we have just seen, considers that the miliary nodules are developed as tuberculous granulations, not in the epithelium, but in the connective tissue.

M. Villemin, more precise, has demonstrated that very often these same nodules result from the proliferation of the model of the connective tissue. inclosed within the alveoli.

We see, therefore, nodules, or rather cellular proliferations which are developed in the same anatomical elements as tuberculous granulations, which to the naked eye differ only in their greater size, which cannot be separately distinguished by the microscope, and still are not identical. When I have added that, according to Lebert, true tubercle is a granulation composed of a proliferation in all respects similar to inflammatory, non-suppurative hyperplasia of the connective tissue, I believe I may be permitted not to know upon what a distinction is based.

*The nodules and granulations may, then, be considered to be two forms of one and the same process.*

Is it necessary for us, on this account, to admit, with M. Villemin, that all the caseous products are the result of the transformation of granulations, of the inspissation followed by the caseous metamorphosis of pus remaining in the pulmonary tissue? I think not.

While the tuberculous granulations are forming there is irritation of all the connective and epithelial elements which are found in their neighborhood; to use an expression of Lebert's, an alveolitis is developed. The vesicular epithelium, if there is such a thing, proliferates; but there is, very surely, hyperplasia of the epithelium of the terminal bronchi, which increase and multiply towards the alveoli, and end by filling them.

The opinion which I am tempted to adopt is, then, neither that of M. Villemin, which seems to me to have restricted too much the part of the bronchial epithelium, nor that of MM. Herard and Cornil, who derive the caseous products, for the most part, from the hypergenesis of the cells of the vesicular epithelium.

But this being understood, I can nevertheless associate myself with these latter authors in saying that *pulmonary phthisis is both connective and epithelial.*

The coincidence of caseous products and granulations is so frequent that Niemeyer, obliged to recognize it, doos not regard it as accidental, but is forced to admit a *causal* relation between the two. But, according to him, in the majority of cases, tubercles are developed tardily, and only complicate pulmonary phthisis at an advanced period. This has led him to the conclusion, somewhat unexpected, that pulmonary phthisis cannot, therefore, be considered as a tubercular phthisis properly so called, notwithstanding the presence of tubercules in the lung. Badoky partakes of this opinion, and also thinks that generally the caseous masses are primitive, and that they provoke the appearance of tubercles.

Niemeyer, however, furnishes us the argument whereby to combat himself. He could not fail to remark the singular distribution of tubercles which are most frequently found exclusively situated in the immediate neighborhood of caverns and caseous deposits. If the deductions, then, which we have just drawn have been followed with attention, it will be instantly seen that this fact alone is sufficient to clear up the whole question. In fact, the inflammatory miliary nodules not being distinguishable from true tuberculous granulations, these nodosities, when they have undergone fatty transformation, can no longer be distinguished from the caseous products resulting from a chronic pneumonic process, and it seems to me perfectly simple and rational to admit that the peripheric granulations are the latter, and nothing more.

The best proof, says M. Colin, that pulmonary phthisis, taken in the largest acceptation of the word given to it by Laennec, is of the same family as granular phthisis, is, that in cases where pulmonary phthisis becomes acute (galloping consumption,) we always find about the caverns and tuberculous masses a crop of gray granulations identically like those of acute tuberculization.

Dr. Bidlot, of Liege, has described, in a recent work, under the name of *accidental or false phthisis*, a consumption, characterized, according to him, by inflammatory pseudo-tubercles. This kind of phthisis should be perfectly separated from *real* phthisis resulting from the evolution of true neoplastic tubercles.

But if, according to this author, pseudo-tubercles cannot be distinguished from true tubercles when they have both undergone caseous transformation, it is only during the first period of phthisis that it is possible to recognize many species of it.

Besides, accidental phthisis, in consequence of the prolonged deterioration of the economy, occasions, most frequently according to M. Bidlot, the production of neoplastic tubercles, whilst the true tubercles of real phthisis induce inflammatory irruptions, the exudations from which are transformed into false tubercles.

When I shall have recalled that even before the period of softening, the distinction between the true granulation and the inflammatory miliary nodule (pseudo-tubercle) has been declared impossible by the ablest micrographists, it seems to me that I am authorized in not accepting the divisions adopted by the physician of Liege. Consequently, if the anatomical demonstration of the preëxistence of tubercular granulation is not always possible, still no fact seems unfavorable to this opinion.

Finally, MM. Herard and Cornil think that the special pneumonia which accompanies the granulations plays the principal rôle in consumption, and it is necessary to attribute to it almost entirely the more or less rapid and more or less extended destruction of the pulmonary tissue.

This theory, very plausible, but which is for its authors themselves only a gratuitous supposition, shou'd have the merit of conciliating opinions and conduct of the unity of the disease.

(To be continued.)

